# Ensuring HbA1c Accuracy and Variant Detection in Hemoglobin G-Coushatta and Queens Using Variant Mode Analysis

**DOI:** 10.3390/diagnostics16091320

**Published:** 2026-04-28

**Authors:** Yeon Jae Lee, Jong Do Seo, Mi-Hyun Hong, Kyunghoon Lee, Joon Hee Lee, Sun-Hee Jun, Hyung-Doo Park, Junghan Song, Yeo-Min Yun

**Affiliations:** 1 Department of Laboratory Medicine, Konkuk University Medical Center, Konkuk University School of Medicine, Seoul 05030, Republic of Korea; yj797@naver.com (Y.J.L.); akame84@hanmail.net (J.D.S.); mati8990@kuh.ac.kr (M.-H.H.); 2 Department of Laboratory Medicine, Seoul National University Bundang Hospital, Seoul National University College of Medicine, Seongnam 13620, Republic of Korea; khlee59023@gmail.com (K.L.); jhleelm@snubh.org (J.H.L.); sunnyeyo@snubh.org (S.-H.J.); 3 Department of Laboratory Medicine and Genetics, Samsung Medical Center, Sungkyunkwan University School of Medicine, Seoul 06351, Republic of Korea; hyungdoo.park@samsung.com

**Keywords:** glycated hemoglobin, Hemoglobin G-Coushatta, Hemoglobin Queens, Arkray ADAMS HA-8190V, Tosoh HLC-723 G11, Bio-Rad D-100, Sebia Capillarys 2 Flex Piercing

## Abstract

**Background/Objectives**: Glycated hemoglobin (HbA1c) is widely used to monitor glycemic control, but the accuracy and flag detection rates of HbA1c assays can vary in the presence of Hb variants such as Hb G-Coushatta (Coushatta) and Hb Queens (Queens), which are common in the Korean population. **Methods**: We evaluated four HbA1c platforms—Arkray ADAMS HA-8190V (HA-8190V) fast/variant modes; Tosoh HLC-723 G11 (G11) standard/variant modes; Bio-Rad D-100 (D-100); Sebia Capillarys 2 Flex Piercing (Capillarys)—using 33 Hb variant samples (26 Coushatta, 7 Queens). The Roche Tina-quant HbA1c Gen. 3 immunoassay was used as the comparative method. With UPLC-MS/MS used as the reference for variant identification, analytical performance was assessed by calculating mean % differences in HbA1c and evaluating Hb variant flag detection rates. **Results**: The mean % differences in HbA1c compared to the comparative method were −1.5% and −0.9% for Coushatta and Queens, respectively, with HA-8190V variant mode; −28.4% and −17.6% with HA-8190V fast mode; +33.5% and +2.2% with the G11 variant mode; −28.1% and −14.3% with G11 standard mode; −5.9% and −3.2% with D-100; and +1.0% and −6.2% with Capillarys. For flag detection, rates were 100% and 0% with HA-8190V fast mode; 84.6% and 28.6% with G11 standard mode; and 100% and 100% with all other platforms. **Conclusions**: Coushatta caused severe underestimation in rapid modes, while Queens hindered automated detection. While variant modes significantly improved detection, they showed platform-dependent accuracy. When Hb variant status is unknown, use of the variant mode is recommended to ensure reliable results.

## 1. Introduction

Glycated hemoglobin (HbA1c) is a key biomarker for assessing long-term glycemic control and is widely used in the diagnosis and management of diabetes [1]. The clinical significance of maintaining optimal HbA1c levels was definitively established by the Diabetes Control and Complications Trial, which demonstrated that intensive glycemic control significantly delays the onset and slows the progression of long-term complications, such as retinopathy and nephropathy [2]. According to the 2026 American Diabetes Association Standards of Care, HbA1c is established as a core diagnostic criterion for diabetes, alongside plasma glucose markers [3]. Accurate measurement is essential to guide clinical decisions, particularly when adjusting therapy or monitoring treatment response. However, hemoglobin (Hb) variants can interfere with HbA1c assays, especially those based on charge separation methods such as high-performance liquid chromatography (HPLC) and capillary electrophoresis, by causing co-elution or incomplete separation and resulting in inaccurate results [4,5,6,7,8,9]. According to the peak separation patterns, these interferences can lead to either spuriously increased or decreased results, primarily determined by the analytical resolution achieved within a given analysis time [8]. In rapid HPLC modes, the shortened elution gradient often provides insufficient time to distinguish between HbA and certain Hb variants. Consequently, if the Hb variant co-elutes with the HbA_0_ fraction, the increased total area of the denominator leads to a falsely decreased HbA1c percentage. Conversely, if the Hb variant or its glycated form comigrates with the HbA1c peak, the numerator increases, resulting in a spurious overestimation [8]. In some cases, the presence of a variant can lead to a spuriously normal HbA1c result due to the misidentification of a variant peak as HbA_0_, which can significantly mask poor glycemic control and delay necessary clinical interventions [10]. The effects of common Hb variants such as HbS, HbC, HbE and HbD are well documented, but the impact of rare Hb variants is less clearly understood [11,12,13,14,15,16].

Among these, Hb G-Coushatta (a β-globin variant) and Hb Queens (an α-globin variant) have been identified as relatively prevalent variants in the Korean population [17,18,19]. Historically, Hb G-Coushatta has been characterized as a frequent variant in populations along the Silk Road, including the Hans and various ethnic minorities in Northwestern China and Central Asia [20,21,22]. An earlier molecular study of 27,006 Korean patients established that these two variants are the most common Hb variants in Korea, with an estimated incidence of 1 in 2700 individuals [17]. Furthermore, a subsequent multi-center study involving four major tertiary referral hospitals in Korea confirmed that these two variants represent a significant portion of the rare Hb variants encountered in clinical practice [18,23]. Beyond their regional presence in Korea, Hb G-Coushatta and Hb Queens are widely distributed throughout East Asia, including China and Southeast Asia, posing a significant diagnostic challenge. A large-scale Chinese study evaluating 14 analytical methods identified these two as among the most frequent regional variants causing clinically significant interference in several HPLC systems [18]. These findings underscore the global clinical relevance of evaluating regional variants to ensure diagnostic integrity in diverse populations. These Hb variants can lead to inaccurate HbA1c values and may not be properly flagged by certain analyzers, increasing the risk of clinical misinterpretation.

A previous study by Yun et al. [19] evaluated the effects of Hb variants using earlier-generation analyzers, including the Tosoh HLC-723 G8 (G8; Tosoh Corporation, Tokyo, Japan) operated in standard mode and the Arkray ADAMS HA-8180 (HA-8180; ARKRAY Inc., Kyoto, Japan) in fast mode. In that study, both the G8 standard mode and HA-8180 fast mode showed substantial negative bias when measuring Hb G-Coushatta and Hb Queens, with over 90% of results exceeding the allowable error margin. Furthermore, flag detection was insufficient; the G8 standard mode failed to flag any Hb Queens cases and missed 51% of Hb G-Coushatta cases, while the HA-8180 fast mode also failed to detect any Hb Queens cases. Since the samples for the present study were sourced from the same major tertiary institutions, these findings are expected to provide highly relevant and longitudinal insights for the management of Korean patients. Similar detection failures and clinically significant biases (exceeding ±10%) have been reported for other variants, such as Hb J-Bangkok and Hb Rambam, when using rapid HPLC modes that prioritize throughput over resolution [12,19].

Since then, updated models have incorporated enhanced detection algorithms. While the earlier HA-8180 was evaluated only in fast mode in previous studies [19], the current Arkray ADAMS HA-8190V (HA-8190V) is evaluated in both fast and variant modes to determine if newer systems offer improved reliability. Similarly, Tosoh HLC-723 G11 (G11) provides a variant mode in addition to the standard mode. Bio-Rad D-100 (D-100; Bio-Rad Laboratories, Inc., Hercules, CA, USA) operates with a single Hb variant-aware analysis mode that includes automated flag detection algorithms. Sebia Capillarys 2 Flex Piercing (Capillarys; Sebia, Lisses, France) uses either visual inspection or system generated alerts based on atypical electrophoretic profiles [12].

In Korean outpatient clinics, shorter turnaround time (TAT) is often prioritized to support efficient patient management. As a result, rapid modes such as Tosoh standard mode and Arkray fast mode are commonly used in routine HbA1c testing, despite potential limitations in Hb variant detection.

This study aimed to evaluate the analytical accuracy and Hb variant flag detection capabilities of four widely used HbA1c analyzers. Two analyzers (Arkray and Tosoh) offer both a rapid and a variant mode. The evaluation focused on Hb G-Coushatta and Hb Queens, which are more frequently encountered in Korean patients. The Roche Tina-quant HbA1c Gen. 3 immunoassay (Roche immunoassay; Roche Diagnostics, Mannheim, Germany) was used as the comparative method. The objective of this study was to assess the measurement accuracy and Hb variant detection reliability of current HbA1c assays in the presence of these clinically relevant Hb variants.

## 2. Materials and Methods

### 2.1. Sample Collection and Hb Variant Identification

A total of 33 clinical samples (26 Hb G-Coushatta and 7 Hb Queens) were exclusively collected at Konkuk University Medical Center (a tertiary referral hospital in Seoul, Korea). These samples were consecutively enrolled during routine HbA1c screening based on the detection of atypical chromatograms or variant flags. All identified variant cases encountered during the study period were included to ensure a comprehensive evaluation of these relatively rare variants. Residual whole blood samples collected in ethylenediaminetetraacetic acid (EDTA) tubes from patients with suspected Hb variants were promptly aliquoted, stored at −70 °C, and transported on dry ice to the participating tertiary laboratories. While samples were handled under standardized protocols, the potential effects of specific storage durations, freeze–thaw cycles, and transport conditions on HbA1c stability and chromatographic resolution were not formally evaluated. To ensure analytical consistency, Hb variant identification was performed at a single central laboratory (Seoul National University Bundang Hospital) using ultra-performance liquid chromatography–tandem mass spectrometry (UPLC-MS/MS) with an LC-30A Nexera UPLC system (Shimadzu Co., Kyoto, Japan) and a Triple Quad 6500 mass spectrometer (Sciex, Framingham, MA, USA) [19]. Additionally, capillary electrophoresis was performed using the Sebia Hb(e) program (Version 9.30) for variant classification. This Hb typing method is distinct from the Capillarys HbA1c program, as it identifies Hb fractions rather than quantifying HbA1c. While samples were handled and transported according to standardized protocols to minimize pre-analytical bias, the potential effects of specific storage durations, freeze–thaw cycles, and transport conditions on HbA1c stability and chromatographic patterns were not formally evaluated in this study. Nonetheless, all aliquots were processed under uniform conditions to maintain sample integrity for comparative analysis.

### 2.2. HbA1c Measurement Assays

All samples were analyzed using four commercial HbA1c assays with two rapid modes, including HA-8190V (HPLC; variant and fast modes), G11 (HPLC; variant and standard modes), D-100 (HPLC), and Capillarys (capillary electrophoresis). The evaluation was conducted across three specialized tertiary hospital laboratories to cover the diverse analytical platforms: the HA-8190V (variant and fast modes) and Capillarys assays were conducted at Konkuk University Medical Center; the G11 standard mode and the D-100 assays at Seoul National University Bundang Hospital; and the G11 variant mode at Samsung Medical Center. Notably, while the measurements for the HPLC platforms (Arkray, Tosoh, and Bio-Rad) were specifically performed for this study, the results for the Capillarys assay were obtained from the initial confirmatory testing performed at the time of sample collection. All instruments were operated following the manufacturers’ instructions and routine quality control. Notably, since no official manufacturer claims exist regarding analytical interference from Hb G-Cushatta and Hb Queens for these platforms, their performance was evaluated independently in this study.

### 2.3. Comparative HbA1c Method

The Roche Tina-quant HbA1c Gen. 3 assay was employed as the comparative method. This assay is based on a competitive turbidimetric inhibition immunoassay (TINIA) principle [24,25]. The specific antibodies used in this reagent target the first four amino acids (valine-histidine-leucine-threonine) at the N-terminal of the β-globin chain, specifically recognizing the glycated N-terminal valine [25,26].

According to the manufacturer’s scientific specifications, interference in this immunoassay is primarily expected when mutations occur within these first four amino acids of the β-chain, as these can alter the antibody-binding epitope [25]. This is consistent with the findings of Jaisson et al. [15], who demonstrated that immunoassays generally show no interference from Hb variants unless the mutation involves the specific epitope recognized by the antibody. In this study, the investigated variants were Hb G-Coushatta [NM_000518.4(HBB):c.68A>C, p.Glu23Ala] and Hb Queens [NM_000558.5(HBA1):c.104T>G, p.Leu35Arg] [19,25]. Since Hb G-Coushatta involves a substitution at the 22nd residue of the β-chain and Hb Queens is an α-chain variant, neither affects the N-terminal sequence of the β-chain targeted by the Roche assay [25,26].

Previous studies have shown that the Roche immunoassay demonstrates strong correlation with HPLC and affinity chromatography methods, with minimal interference from common Hb variants such as HbS, HbC, HbD, and HbE [7,24,27]. Notably, the robustness of immunoassay platforms against interference from various Hb variants, such as HbE, HbD, and even rarer types like Hb G-Taichung and Hb Wayne has been well-documented across multiple comparative studies [5,12,28,29,30]. Given that the structural modifications in Hb G-Coushatta and Hb Queens occur at positions distant from the N-terminal glycation site, they were not expected to interfere with the immunoassay results. Therefore, the Roche immunoassay was considered an appropriate comparative method for evaluating the analytical performance of the other assays in the presence of these clinically relevant Hb variants [19,25].

### 2.4. Flag Detection Evaluation for Hb Variants

Each assay’s ability to detect Hb variants was evaluated by recording any automated flags or system-generated comments indicating abnormal chromatographic or electrophoretic patterns. The detection rate was defined as the percentage of samples correctly flagged by each assay among the total number of variant cases confirmed by the reference method, UPLC-MS/MS. Detection rates were calculated separately for each assay and Hb variant group.

### 2.5. Data Analysis

HbA1c results from each assay mode were compared with the immunoassay values. The mean % difference in HbA1c was calculated using the following formula:

Mean % difference in HbA1c = (Assay HbA1c − Roche Immunoassay HbA1c)/Roche Immunoassay HbA1c × 100

Positive values indicated overestimation, while negative values indicated underestimation relative to the immunoassay. Mean % differences in HbA1c were calculated separately for each assay and Hb variant type. To evaluate the analytical performance, we adopted a total allowable error (TEa) of ±2.4%, which corresponds to the desirable specification based on the European Federation of Clinical Chemistry and Laboratory Medicine (EFLM) biological variation database [31]. Agreement between methods was further assessed using Bland–Altman plots, generated with MedCalc Statistical Software version 23.1.5 (MedCalc Software Ltd., Ostend, Belgium). Summary statistics and graphical visualizations were prepared using Microsoft Excel 2019 (Microsoft Corp., Redmond, WA, USA).

## 3. Results

### 3.1. Hb Variant Identification and Electrophoresis Findings

**Figure 1:**
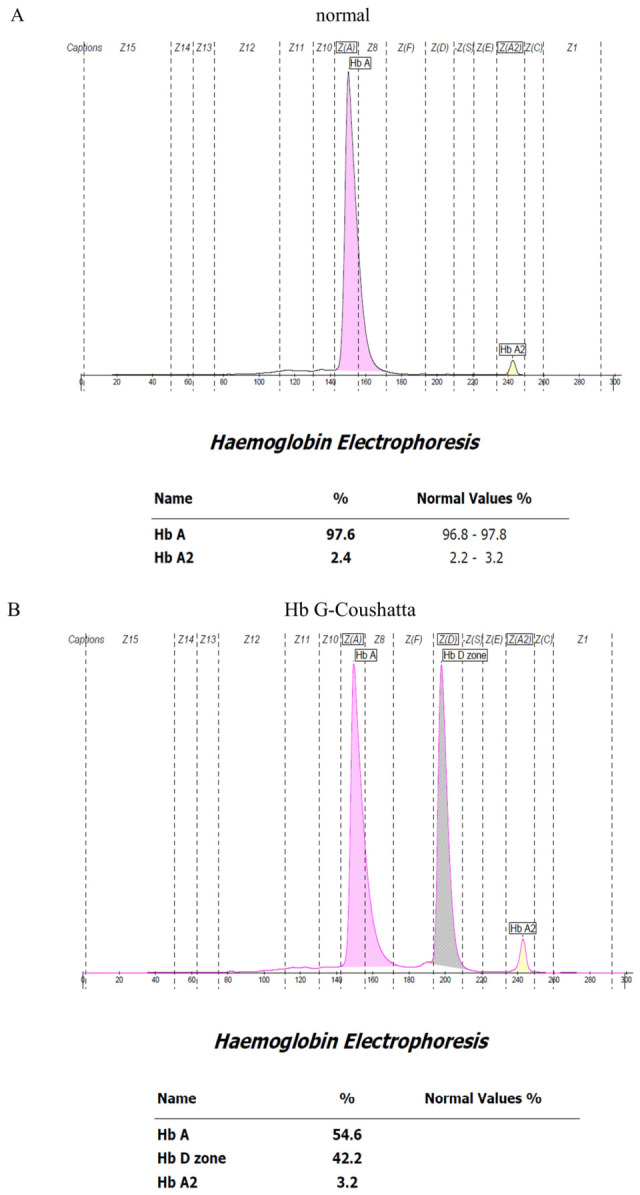

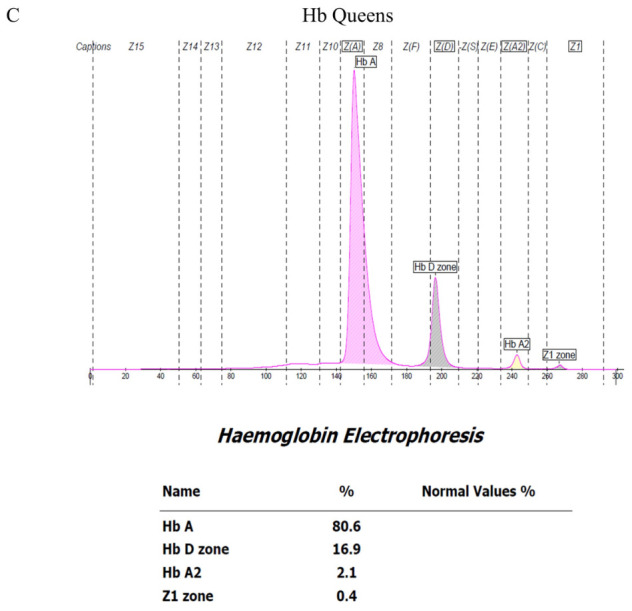
Representative Hb EP patterns in control and each Hb variant samples. (**A**) Normal control, (**B**) Hb G-Coushatta, and (**C**) Hb Queens.

Among the 33 samples confirmed by UPLC-MS/MS as the reference method (26 Hb G-Coushatta and 7 Hb Queens), Hb electrophoresis was additionally performed to assess the distribution of Hb fractions (Figure 1). All samples showed distinctive Hb variant profiles consistent with their molecular classification (Table 1).

### 3.2. Analytical Accuracy (Mean % Difference in HbA1c)

**Figure 2:**
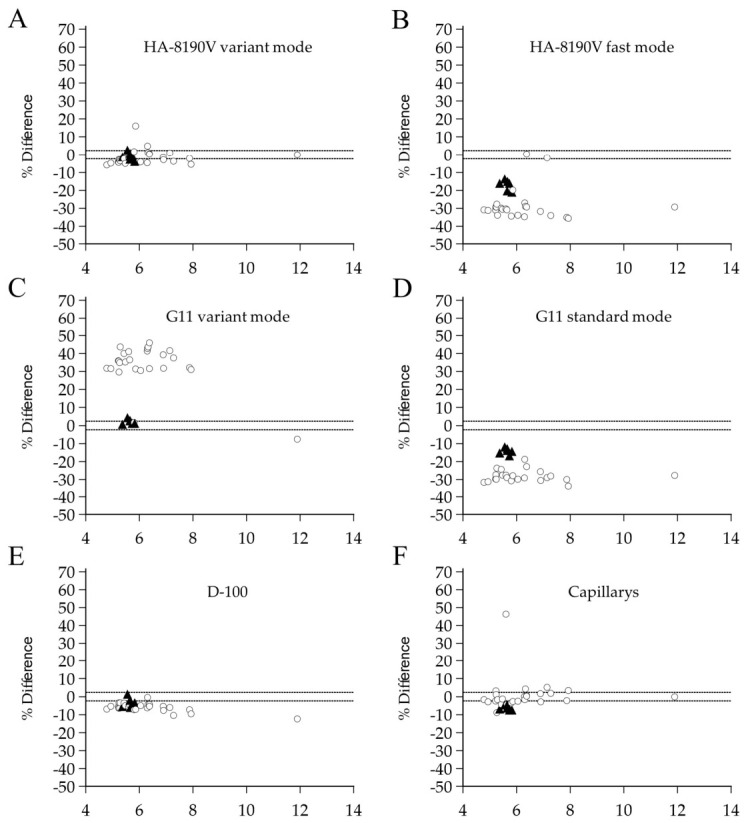
Bland–Altman plots showing the mean % difference in HbA1c measurements between each assay and The Roche Tina-quant HbA1c Gen. 3 immunoassay in 33 samples with Hb G-Coushatta and Hb Queens variants. (**A**) HA-8190V variant mode; (**B**) HA-8190V fast mode; (**C**) G11 variant mode; (**D**) G11 standard mode; (**E**) D-100; and (**F**) Capillarys. The *x*-axis represents the HbA1c values obtained from the Roche immunoassay as the comparative method. The mean % difference in HbA1c was calculated as (Assay HbA1c − Immunoassay HbA1c)/Immunoassay HbA1c × 100. Each point represents an individual sample. The dashed lines represent the EFLM desirable total allowable error limit of ±2.4%. Open circles indicate samples with Hb G-Coushatta, and black triangles indicate those with Hb Queens. Hb variant types were confirmed using UPLC-MS/MS. Abbreviations: HbA1c, Glycated Hemoglobin; UPLC-MS/MS, Ultra-Performance Liquid Chromatography–Tandem Mass Spectrometry; EFLM, European Federation of Clinical Chemistry and Laboratory Medicine; HA-8190V, Arkray ADAMS HA-8190V (Arkray, Kyoto, Japan); G11, Tosoh HLC-723 G11 (Tosoh, Tokyo, Japan); D-100, Bio-Rad D-100 (Bio-Rad, Hercules, CA, USA); Capillarys, Sebia Capillarys 2 Flex Piercing (Sebia, Lisses, France).

Overall, analytical performance varied significantly across platforms and modes in a platform-dependent fashion. Although the National Glycohemoglobin Standardization Program (NGSP) currently uses a ±5.0% criterion for manufacturer certification, we evaluated analytical interference based on the EFLM biological variation database, which is a higher-priority specification according to Clinical and Laboratory Standards Institute (CLSI) EP21 guidelines. When applying the EFLM desirable TEa threshold of ±2.4%, most assays demonstrated high interference rates for at least one variant. Detailed mean % differences, 95% CIs, and interference frequencies are summarized in Table 2 and visualized in Figure 2.

The HA-8190V variant mode (Figure 2A) showed the smallest mean % differences (−1.5% for Hb G-Coushatta; −0.9% for Hb Queens). However, interference was still identified in 61.5% and 42.9% of samples, respectively, as many points fell outside the ±2.4% limits. In contrast, the rapid analysis modes (HA-8190V fast, Figure 2B; G11 standard, Figure 2D) showed unacceptable accuracy due to marked underestimation (range: −28.4% to −14.3%). Interference occurred in nearly all samples (92.3–100%), including cases with no reportable results.

The G11 variant mode (Figure 2C) exhibited variant-specific interference. While it showed a relatively small mean bias for Hb Queens (+2.2%; 95% CI: 1.2% to 3.1%), more than half of these samples (57.1%) exceeded the ±2.4% interference threshold. In contrast, for Hb G-Coushatta, the assay demonstrated a substantial positive mean % difference of +33.5% (95% CI: 29.0% to 38.1%), with interference observed in nearly all samples (96.2%; 25/26).

The D-100 (Figure 2E) maintained a consistent negative bias (−5.9% for Hb G-Coushatta; −3.2% for Hb Queens), resulting in interference rates of 96.2% and 57.1%, respectively. Capillarys (Figure 2F) demonstrated the widest individual variability for Hb G-Coushatta (mean +1.0%, but 50.0% interference), while all Hb Queens samples (100.0%) were consistently underestimated beyond −2.4% (mean −6.2%).

Across the measured range (4.0–12.0%), the percentage bias for most assays remained relatively constant. No significant concentration-dependent patterns were observed, and the variability of the bias was uniform across all HbA1c levels. This suggests that the interference from these variants is primarily independent of the underlying glycated Hb concentration.

### 3.3. Flag Detection Performance for Hb Variants

Flag detection rates varied significantly across assays and Hb variant types (Table 3). The HA-8190V variant mode, G11 variant mode, and D-100 correctly flagged 100% of samples for both Hb G-Coushatta and Hb Queens. In contrast, the G11 standard mode failed to detect 4 cases of Hb G-Coushatta (4/26, 15.4%) and 5 cases of Hb Queens (5/7, 71.4%). The HA-8190V fast mode correctly flagged all Hb G-Coushatta samples but failed to detect all 7 cases of Hb Queens (0/7, 0.0%). Detailed analytical characteristics, including the specific types and frequencies of flags reported by each assay, are summarized in Table S1.

## 4. Discussion

This study evaluated the analytical performance and Hb variant-flag detection of four HbA1c assays, including the HA-8190V in fast and variant modes, the G11 in standard and variant modes, D-100, and Capillarys. The evaluation focused on Hb G-Coushatta and Hb Queens, which are relatively prevalent in the Korean population [17,18,19]. Beyond their regional presence in Korea, these variants are widely distributed throughout East Asia, particularly in China and Southeast Asia, representing a significant diagnostic challenge in global health. For instance, a comprehensive Chinese study evaluating seven hemoglobin variants across 14 analytical methods highlighted that Hb G-Coushatta and Hb Queens are among the most frequently encountered variants in the region, often leading to clinically significant interference in several HPLC systems [18]. All 33 samples were confirmed by UPLC-MS/MS and electrophoresis, confirming that capillary electrophoresis combined with MS-based methods provides reliable classification of these variants.

Significant differences in analytical accuracy were observed depending on assay type and mode. The HA-8190V variant mode demonstrated the best overall agreement with the immunoassay reference, with mean % differences within ±2.4% for both variants, and successfully flagged all cases. These findings align with Shin et al. [32], who reported minimal biases of 2.63% for Hb G-Coushatta and 3.65% for Hb Queens in this mode. While Song et al. [23] also reported a comparable bias of 5.0% for Hb G-Coushatta, our study provides additional validation for Hb Queens, which was not addressed in their evaluation, further confirming the effectiveness of this mode in mitigating interference from both variants. Conversely, the HA-8190V fast mode consistently underestimated HbA1c values, particularly for Hb G-Coushatta (−28.4%). Furthermore, it demonstrated limited flag detection, failing to flag any Hb Queens cases. This mirrors the negative bias (−12.5%) reported by Song et al. [23] and underscores the risk of clinical misinterpretation when using shortened gradients.

The G11 also exhibited mode-dependent performance. The G11 variant mode performed well for Hb Queens (+2.2%), consistent with the nearly zero bias reported by Park et al. [33]. However, it showed a substantial positive bias for Hb G-Coushatta (+33.5%), which is significantly higher than the +12.1% previously reported for the same model [33]. In contrast, the G11 standard mode showed significant underestimation for both variants (−28.1% for Hb G-Coushatta and −14.3% for Hb Queens). These findings are highly consistent with previous reports for Hb G-Coushatta (−30.3% to −31.8%) and directly align with the negative biases of −14.4% to −18.5% reported for Hb Queens [32,33]. Crucially, while the variant mode flagged all samples, the standard mode failed to flag 71.4% of Hb Queens cases, reinforcing the risk of reporting clinically misleading HbA1c values without any flag.

The D-100 relatively improved performance compared to rapid HPLC modes, although the mean bias for both variants exceeded the EFLM desirable total allowable error limit (±2.4%). Our observed bias for Hb G-Coushatta (−5.9%) is comparable to the −4.1% reported by Song et al. [23]. Notably, our study provides new performance data for Hb Queens on the D-100 platform, which showed a mean bias (−3.2%) and was not covered in previous study [23]. The assay successfully identified flags for all samples, confirming its utility in detecting these specific Korean variants without the severe interference seen in rapid HPLC modes.

The Capillarys results were variable but generally accurate. For Hb G-Coushatta, our observed bias (+1.0%) aligns well with previous reports of +0.9% to +3.0% [19,23]. However, for Hb Queens, we observed a mild underestimation (−6.2%), which is slightly more pronounced than the −2.1% bias reported by Yun et al. [19]. Despite these minor variations in accuracy, Capillarys demonstrated high clinical utility by successfully flagging all Hb variants, ensuring that potential interferences are recognized during routine screening.

Analytical performance differences among platforms primarily stem from their separation principles and analysis times. Capillary electrophoresis is less susceptible to interference because its extended analysis time provides superior resolution to distinguish variants. In contrast, for HPLC-based platforms, while reagent composition and peak morphology play roles, the elution gradient duration is the most critical factor. Rapid HPLC modes often lack the resolution necessary to separate subtle regional variants like Hb G-Coushatta or Hb Queens. This shortened analysis time is the major cause of the significant biases and failed flag detections observed in ‘fast’ or ‘standard’ modes compared to variant-optimized modes. Our findings gain clinical significance when compared to HbS and HbC, the most prevalent β-chain variants. While HbS and HbC show classic migration in the S and C zones on Hb electrophoresis, Hb G-Coushatta (β22 Glu → Ala) exhibits a distinct peak, reflecting its unique charge alteration. Furthermore, Hb Queens (α34 Leu → Arg) is an α-chain variant, distinguishing it from the β-subunit mutations of HbS/C. Since α-variants can affect all hemoglobin fractions (HbA, A2, and F), they present more complex interference patterns. Beyond analytical interference, the clinical recognition of Hb variants is vital because certain mutations can lead to functional abnormalities. For instance, variants such as Hb M-Ratnagiri (β 63 His → Tyr) or mutations in the NADH-cytochrome b_5_ reductase gene have been associated with congenital methemoglobinemia and severe clinical manifestations, including intellectual disability [34,35]. While the variants in our study (Hb G-Coushatta and Hb Queens) are primarily associated with HbA1c interference rather than abnormal oxygen transport, these examples highlight the broader diagnostic responsibility of laboratories to identify and investigate unexplained hemoglobin patterns that may signify underlying functional hemoglobinopathies.

The flag detection performance and the direction of bias were closely linked to the analytical resolution of each assay, as evidenced by the chromatograms in Supplementary Figures S1–S3. In the HA-8190V fast mode and G11 standard mode, both Hb G-Coushatta and Hb Queens exhibited patterns where the Hb variant co-eluted with the HbA_0_ fraction while the glycated Hb variant separated from HbA1c. This co-elution increases the denominator in the HbA1c calculation, leading to the observed spurious decrease (negative bias) in results [8]. In contrast, platforms with higher resolution and extended elution gradients, including HPLC-based systems such as the HA-8190V variant mode and D-100 as well as the capillary electrophoresis-based Capillarys, demonstrated that both Hb variant and glycated Hb variant were fully separable from HbA and HbA1c, respectively [8]. This superior resolution allowed for accurate HbA1c determination and reliable flagging. Interestingly, the G11 variant mode showed divergent patterns: while Hb Queens was fully separated, Hb G-Coushatta exhibited a pattern where the glycated Hb variant comigrated with HbA1c while the Hb variant remained separable from HbA_0_ [8]. This comigration, potentially exacerbated by the use of frozen samples [36,37], likely produced the observed significant positive bias for Hb G-Coushatta on this platform. These findings underscore that while variant-optimized modes generally offer higher resolution than short-gradient rapid modes, the degree of interference remains variant-specific depending on the specific elution characteristics of each platform.

Our results emphasize that relying solely on the absence of automated flag alerts is insufficient to ensure analytical accuracy, particularly in high-throughput settings using rapid HPLC modes. The clinical risk is particularly pronounced with variants like Hb Queens, which frequently escape detection in shortened gradients, potentially leading to the mismanagement of diabetic patients due to underestimated HbA1c values. Therefore, laboratories in regions where Hb G-Coushatta and Hb Queens are prevalent should prioritize analytical platforms with superior resolution, such as variant-optimized HPLC or capillary electrophoresis. When results are clinically discordant or exhibit unexpected analytical deviations, proactive confirmatory testing using alternative methodologies like immunoassays is essential to guarantee diagnostic integrity.

This study has several limitations. First, the total sample size was small (*n* = 33) and imbalanced (Hb G-Coushatta, *n* = 26 vs. Hb Queens, *n* = 7). This disparity, particularly for Hb Queens, limits the statistical power of our analysis and may affect the reliability of the observed trends. Consequently, findings for Hb Queens should be interpreted with caution as they may not fully represent the broader population of this variant. Further studies with larger, more balanced cohorts are necessary to enhance statistical precision and confirm the generalizability of our conclusions. Second, while this study focuses on two Hb variants prevalent in Korea, the findings may not be extrapolated to other rare or compound Hb variants, nor to non-Korean populations. Since the interference of Hb variants on HbA1c assays can be ethnic-specific, further studies involving diverse ethnic groups and a broader range of Hb variants are necessary to confirm these results globally. Third, although Hb variants were identified using UPLC-MS/MS, the lack of genetic confirmation via DNA sequencing is a limitation. While UPLC-MS/MS is highly sensitive for detecting amino acid substitutions, genetic testing would have provided definitive evidence and eliminated any potential risk of misclassification. Additionally, while a Roche immunoassay served as the comparative method, further validation using additional gold-standard methodologies could further solidify these results. Fourth, a Roche immunoassay was used as the comparative method instead of the IFCC primary reference procedure (HPLC-MS), as samples were not pre-processed for mass spectrometry. However, immunoassays are typically affected only by mutations within the first four amino acids of the β-chain N-terminus [25,26]. Since Hb G-Coushatta (β22) and Hb Queens (α34) are located far from this antibody-binding epitope, the immunoassay remains a scientifically sound baseline. Nevertheless, the absence of a primary reference method is a limitation, and future studies using IFCC-standardized methodologies are warranted to definitively validate these findings. Fifth, the potential impact of pre-analytical variables remains a limitation. Although samples were stored at −70 °C and transported under standardized cold-chain conditions, the lack of matched fresh samples prevented a systematic evaluation of storage-induced effects. Specific stressors, such as freeze–thaw cycles and prolonged storage, can induce minor protein modifications or aggregation, which may subtly alter chromatographic elution profiles in highly sensitive HPLC modes [36,37]. This may have contributed to the unexpectedly large bias observed in the G11 variant mode, and future studies using fresh samples are required to isolate true analytical interference from storage-induced artifacts. Seventh, a fundamental constraint of this study is the inability to export raw spectral or integration data, as clinical-grade HbA1c analyzers are designed for automated result reporting rather than raw data re-analysis. Consequently, our assessment relied on vendor-provided chromatograms and flags. To supplement this, representative chromatograms and electropherograms are provided in Supplementary Figures S1–S3 for qualitative evaluation of elution profiles and peak separation. Furthermore, while testing was conducted at three institutions, most analyses were performed at a single site. Multicenter studies are needed to validate these findings in broader clinical settings and populations. In addition, although we hypothesized that pre-analytical factors contributed to the G11 variant mode’s positive bias, the lack of matched fresh samples prevented a definitive conclusion [14], but further investigation is warranted. This limitation may affect the interpretation of results obtained using the Tosoh variant mode for this specific Hb variant.

## 5. Conclusions

This study demonstrates that Hb variants significantly impact HbA1c accuracy and detection performance according to the analytical platform and mode. Hb G-Coushatta caused severe underestimation in rapid HPLC modes, while Hb Queens frequently escaped automated detection, particularly in the HA-8190V fast and G11 standard modes. In contrast, the variant modes of both HA-8190V and G11, the D-100, and the Capillarys platforms achieved 100% flag detection for both variants, ensuring higher diagnostic reliability.

Given the prevalence of these variants in the Korean population, clinical laboratories should prioritize the use of variant modes or capillary electrophoresis for initial testing. Furthermore, as the absence of a flag alert does not always guarantee accuracy, results that are clinically discordant should be confirmed using alternative methodologies to prevent the potential mismanagement of diabetic patients.

## Figures and Tables

**Table 1:** Distribution of Hb variants in samples with abnormal electrophoresis patterns (*n* = 33) *.

Hb EP †		Hb Variant	Codon and Amino Acid Change	*N* (%)
Pattern	Interpretation			
Z6 (D zone)	β variant	Hb G-Coushatta	NM_000518.4(HBB):c.68A>C, p.Glu23Ala	26 (78.8)
Z6 (D zone) + Z1	α variant	Hb Queens	NM_000558.5(HBA1):c.104T>G, p.Leu35Arg.	7 (21.2)

* All 33 samples showing abnormal Hb EP patterns suspicious for Hb variants were confirmed by UPLC-MS/MS or gene sequencing. † Hb EP was performed using the Capillarys 2 Flex Piercing system (Sebia, Lisses, France). Abbreviations: Hb EP, Hemoglobin Electrophoresis; UPLC-MS/MS, Ultra-Performance Liquid Chromatography–Tandem Mass Spectrometry.

**Table 2:** Mean % difference in HbA1c and interference of HbA1c assays in samples with Hb G-Coushatta and Hb Queens variants (*n* = 33).

Methods	Hb G-Coushatta * (*n* = 26)	Hb Queens * (*n* = 7)	Interference † (Hb G-Coushatta)	Interference † (Hb Queens)
HA-8190V variant mode	−1.5 (−3.2, 0.2) ‡	−0.9 (−2.4, 0.7) ‡	3/26 (11.5)	0/7 (0.0)
HA-8190V fast mode	−28.4 (−31.9, −25.0)	−17.6 (−19.8, −15.4)	24/26 (92.3) §	7/7 (100.0)
G11 variant mode	33.5 (29.0, 38.1)	2.2 (1.2, 3.1) ‡	25/26 (96.2)	0/7 (0.0)
G11 standard mode	−28.1 (−29.4, −26.9)	−14.3 (−15.5, −13.0)	26/26 (100.0) §	7/7 (100.0) §
D-100	−5.9 (−6.9, −5.0)	−3.2 (−5.1, −1.3)	18/26 (69.2)	2/7 (28.6)
Capillarys	1.0 (−2.8, 4.7) ‡	−6.2 (−7.1, −5.3)	3/26 (11.5)	5/7 (71.4)

* Values are presented as mean % difference (95% confidence interval). Mean % difference in HbA1c was calculated as: (Assay HbA1c − Roche Immunoassay HbA1c)/Roche Immunoassay HbA1c × 100. † Interference was defined as either a mean % difference in HbA1c beyond ±2.4% (EFLM desirable performance specification for bias) or no reportable result. Values for interference are presented as number (percentage). ‡ Met the desirable performance specification for bias (±2.4%) derived from the TEa limits of the EFLM biological variation database. § In HA-8190V fast mode, no reportable result was obtained for one Hb G-Coushatta sample. For Tosoh G11 standard mode, no reportable results were obtained for three Hb G-Coushatta samples and one Hb Queens sample. Abbreviations: EFLM, European Federation of Clinical Chemistry and Laboratory Medicine; Hb, hemoglobin; HbA1c, glycated hemoglobin; TEa, total allowable error; HA-8190V, Arkray ADAMS HA-8190V (Arkray, Kyoto, Japan); G11, Tosoh HLC-723 G11 (Tosoh, Tokyo, Japan); D-100, Bio-Rad D-100 (Bio-Rad, Hercules, CA, USA); Capillarys, Sebia Capillarys 2 Flex Piercing (Sebia, Lisses, France).

**Table 3:** Detection rates (%) of Hb variant flags * by HbA1c assays in Hb G-Coushatta and Hb Queens variants (*n* = 33).

Method	Hb G-Coushatta (*n* = 26)	Hb Queens (*n* = 7)
HA-8190V variant mode	100% (26)	100% (7)
HA-8190V fast mode	100% (26)	0% (0)
G11 variant mode	100% (26)	100% (7)
G11 standard mode	84.6% (22)	28.6% (2)
D-100	100% (26)	100% (7)
Capillarys	100% (26)	100% (7)

* A sample was considered “flagged” when the instrument reported an alert, message, or abnormal chromatographic/electrophoretic pattern indicating the presence of an Hb variant. Values are presented as the percentage and number of samples with successful flag detection. The Arkray ADAMS HA-8190V variant mode, Bio-Rad D-100, and Sebia Capillarys 2 Flex Piercing flagged all samples (100%) from both Hb variant groups. In contrast, the Arkray ADAMS HA-8190V fast mode flagged all Hb G-Coushatta samples but none of the Hb Queens samples (0%), while the Tosoh HLC-723 G11 standard mode showed lower flag detection rates in both variant groups. Abbreviations: HbA1c, Glycated Hemoglobin; N, number; HA-8190V, Arkray ADAMS HA-8190V (Arkray, Kyoto, Japan); G11, Tosoh HLC-723 G11 (Tosoh, Tokyo, Japan); D-100, Bio-Rad D-100 (Bio-Rad, Hercules, CA, USA); Capillarys, Sebia Capillarys 2 Flex Piercing (Sebia, Lisses, France).

## Data Availability

The datasets generated during and/or analyzed during this study are not publicly available due to restrictions imposed by the institutional review board (IRB), which does not permit the transfer of raw data outside the research institution.

## Supplementary Materials

The following supporting information can be downloaded at: https://www.mdpi.com/article/10.3390/diagnostics16091320/s1, Table S1: Detection rates (%) of Hb variant flags * by HbA1c assays in Hb G-Coushatta and Hb Queens variants (*n* = 33); Table S2: Categorical Agreement of HbA1c Measurements based on the Clinical Diagnostic Threshold (6.5%); Figure S1: The chromatograms or electropherograms of normal control sample by Arkray HA-8190V variant/fast mode, Tosoh HLC-723 G11 variant/fast mode, Bio-Rad D-100, and Sebia Capillarys 2 Flex Piercing; Figure S2: The chromatograms or electropherograms of the Hb G Coushatta sample by Arkray HA-8190V variant/fast mode, Tosoh HLC-723 G11 variant/fast mode, Bio-Rad D-100, and Sebia Capillarys 2 Flex Piercing; Figure S3: The chromatograms or electropherograms of the Hb Queens sample by Arkray HA-8190V variant/fast mode, Tosoh HLC-723 G11 variant/fast mode, Bio-Rad D-100, and Sebia Capillarys 2 Flex Piercing; Figure S4: Passing–Bablok regression analysis of HbA1c measurement methods in 33 samples with Hb G-Coushatta and Hb Queens variants.
