# Supplementary material for: Ensuring HbA1c Accuracy and Variant Detection in Hemoglobin G-Coushatta and Queens Using Variant Mode Analysis
Source: Diagnostics (Basel). 2026 Apr 28;16(9):1320. doi: 10.3390/diagnostics16091320 (PMC13163505; doi:10.3390/diagnostics16091320)
Supplement: Supplementary file 1 [file diagnostics-16-01320-s001.zip › diagnostics-4216196-supplementary.pdf]

Supplemental Data

Supplementary Table S1. Detection rates (%) of Hb variant flags* by HbA1c assays in Hb G-Coushatta and Hb Queens variants (N=33)

Method	Hb G-Coushatta (N=26)	Hb Queens (N=7)	Hb G-Coushatta flags (N)	Hb Queens flags (N)
HA-8190V variant mode	100% (26)	100% (7)	Abnormal peak (E) detected (26)	HbS (7)
HA-8190V fast mode	100% (26)	0% (0)	Abnormal peak (E) detected (18)	No flag (7)
			Abnormal peak E (6)	
			Duplex peak (2)	
G11 variant mode	100% (26)	100% (7)	Flag 24 & 40 (24)	Flag 40 (7)
			Flag 40 (1)	

			Flag 24 & 43 (1)	
G11 standard mode	84.6% (22)	28.6% (2)	Flag 24 (16)	Flag 01 (2)
			Flag 02 (3)	No flag (5)
			Flag 01 (2)	
			Flag 01 & 24 (1)	
			No flag (4)	
D-100	100% (26)	100% (7)	Peak in E-Window (18)	Peak in S-Window (7)
			Possible variant interference<br>Elevated peak in E-window (7)	
			Peak in D-Window (1)	

Capillarys	100% (26)	100% (7)	Atypical profile (26)	Atypical profile (7)

* A sample was considered "flagged" when the instrument reported an alert, message, or abnormal chromatographic/electrophoretic pattern indicating the presence of a Hb variant. Values are presented as the percentage and number of samples with successful flag detection.

Tosoh G11 variant mode flags: Flag 24, more than one unknown peak detected; Flag 40, H-VAR peak detected; Flag 43, P-HV3 (HbE type) peak detected.

Tosoh G11 standard mode flags: Flag 01, total area low; Flag 02, HbA1c < 4.0%; Flag 24, variant suspected.

Arkray ADAMS HA-8190V flags: Abnormal peak (E), peak detected in the HbE window; Duplex peak, overlapping or split peak integration; HbS, peak detected in the HbS window.

Bio-Rad D-100 flags: Peak in E-window, peak detected in the HbE window; Peak in D-window, peak detected in the HbD window; Peak in S-window, peak detected in the HbS window.

Abbreviations: Hb, hemoglobin; HbA1c, glycated hemoglobin; N, number; HA-8190V, Arkray ADAMS HA-8190V (Arkray, Kyoto,

Japan); G11, Tosoh HLC-723 G11 (Tosoh, Tokyo, Japan); D-100, Bio-Rad D-100 (Bio-Rad, Hercules, CA, USA); Capillarys, Sebia Capillarys 2 Flex Piercing (Sebia, Lisses, France).

Supplementary Table S2. Categorical Agreement of HbA1c Measurements based on the Clinical Diagnostic Threshold (6.5%)

Diagnostic Category	Diabetes (≥ 6.5%)	Misclassified
Roche immunoassay	7 cases	
HA-8190V variant mode	9 cases	2 Over-diagnosed
HA-8190V fast mode	2 cases	5 Under-diagnosed
G11 variant mode	24 cases	17 Over-diagnosed
G11 standard mode	1 case	6 Under-diagnosed
D-100	6 cases	1 Under-diagnosed
Capillarys	9 cases	2 Over-diagnosed

Categorical agreement was evaluated based on the American Diabetes Association (ADA) diagnostic threshold for diabetes (≥6.5%), using the Roche immunoassay as the reference method for clinical classification.

Abbreviations: HbA1c, glycated hemoglobin; C, Hb G-Coushatta variant; Roche immunoassay, The Roche Tina-quant HbA1c Gen. 3 immunoassay; HA-8190V, Arkray ADAMS HA-8190V (Arkray, Kyoto, Japan); G11, Tosoh HLC-723 G11 (Tosoh, Tokyo, Japan); D-100, Bio-

Rad D-100 (Bio-Rad, Hercules, CA, USA); Capillarys, Sebia Capillarys 2 Flex Piercing (Sebia, Lisses, France).

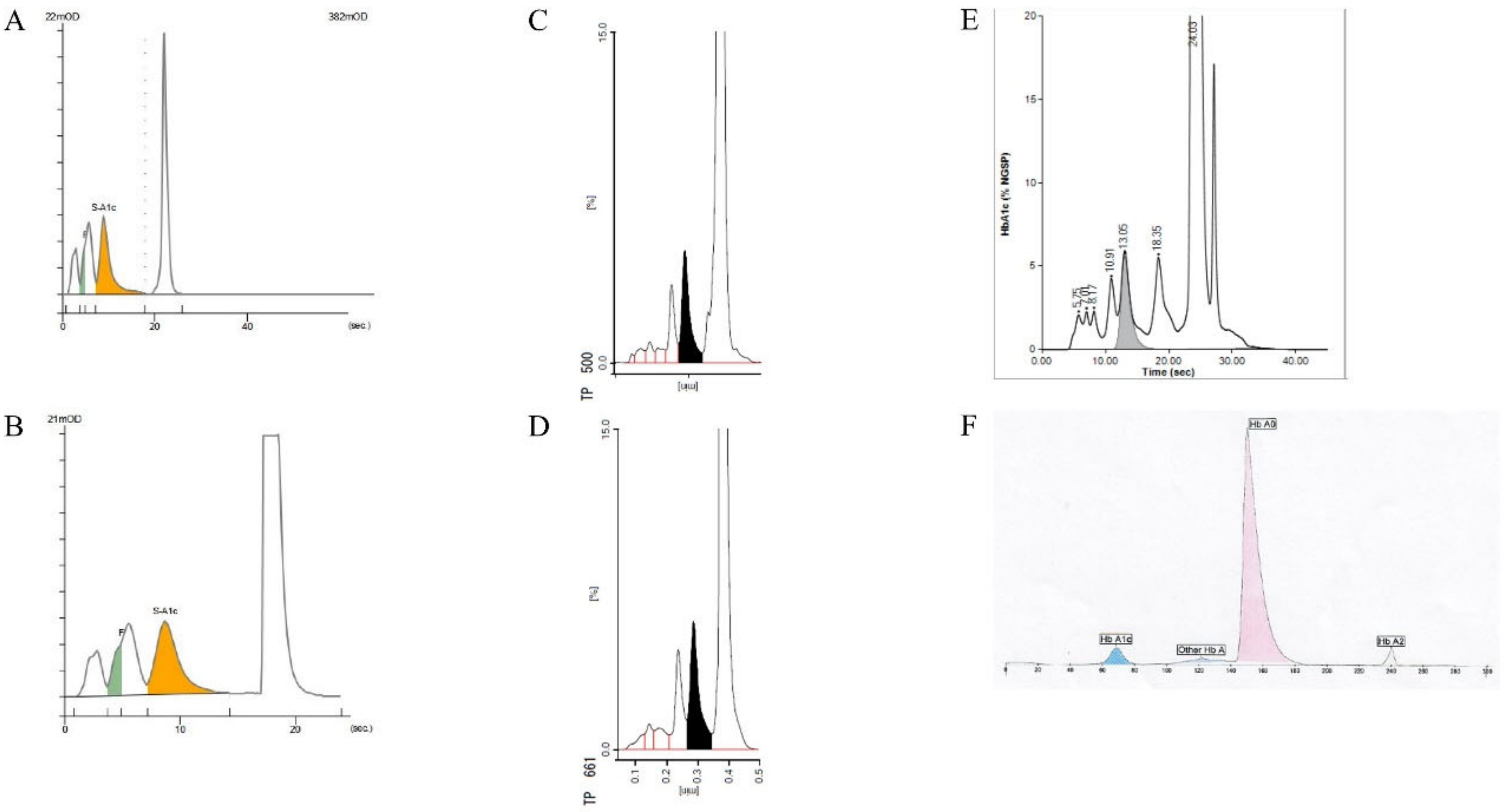
A
22mOD
382mOD
S-A1c
0
20
40
(sec.)
B
21mOD
F
S-A1c
0
10
20
(sec.)
C
15.0
[%]
0.0
TP 500
[mm]
D
15.0
[%]
0.0
TP 661
0.1
0.2
0.3
0.4
0.5
[mm]
E
HbA1c (% NGSP)
20
15
10
5
0
5.75
7.01
8.17
10.91
13.05
18.35
24.03
0.00
10.00
20.00
30.00
40.00
Time (sec)
F
Hb A1c
Other Hb A
Hb A0
Hb A2

Supplementary Figure S1. The chromatograms or electropherograms of normal control sample by Arkray HA-8190V variant/fast mode, Tosoh HLC-723 G11 variant/fast mode, Bio-Rad D-100, and Sebia Capillarys 2 Flex Piercing.

(A) Arkray ADAMS HA-8190V variant mode, (B) Arkray ADAMS HA-8190V fast mode, (C) Tosoh HLC-723 G11 variant mode, (D) Tosoh HLC-723 G11 standard mode, (E) Bio-Rad D-100, (F) Sebia Capillarys 2 Flex Piercing. Abbreviations: Hb, hemoglobin

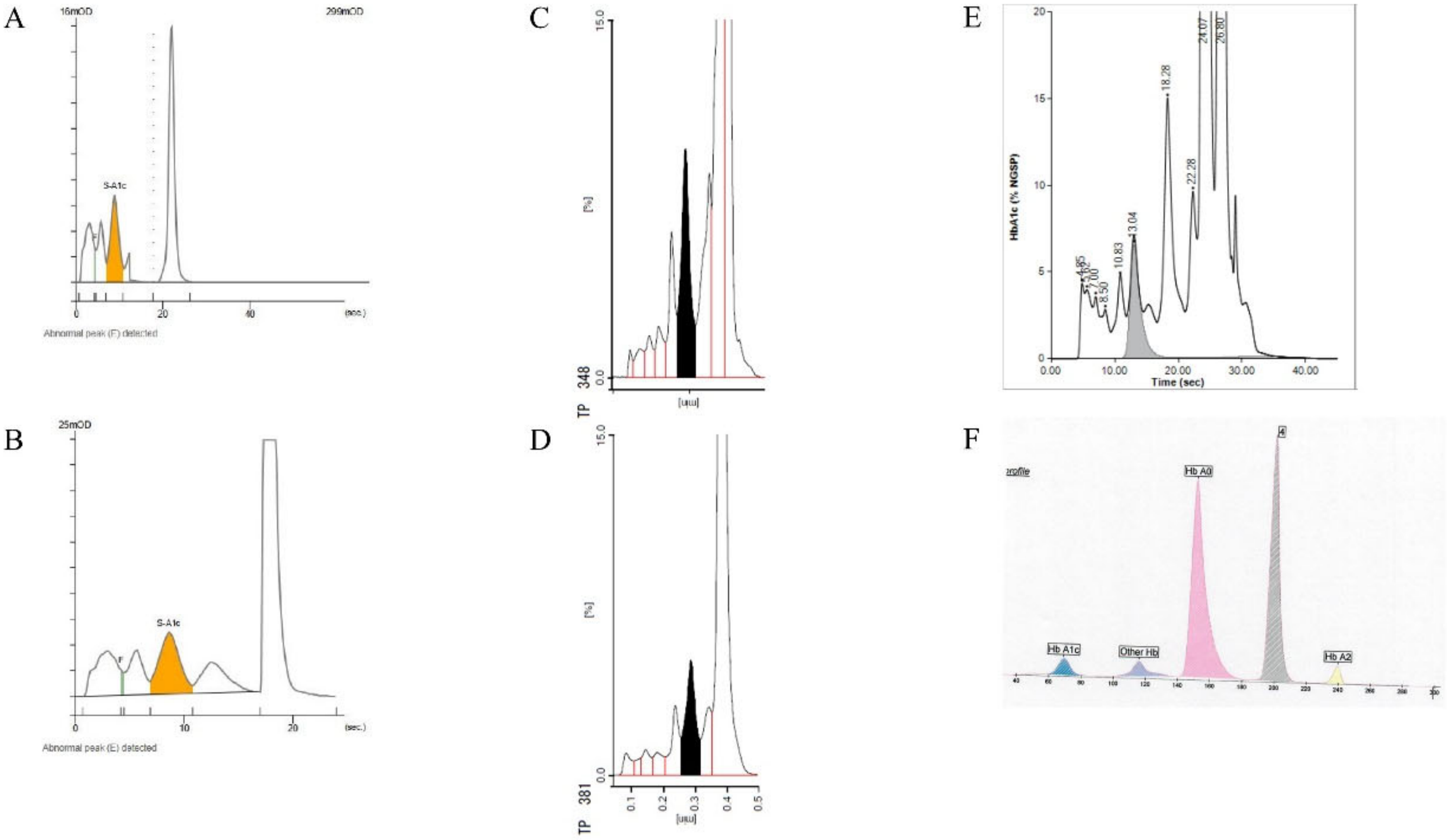
A
16mOD
299mOD
S-A1c
(sec.)
Abnormal peak (F) detected
B
25mOD
S-A1c
(sec.)
Abnormal peak (E) detected
C
15.0
[%]
TP 348
D
15.0
[%]
TP 381
E
HbA1c (% NGSP)
Time (sec)
F
Hb A1c
Other Hb
Hb A0
Hb A2

Supplementary Figure S2. The chromatograms or electropherograms of Hb G Coushatta sample by Arkray HA-8190V variant/fast mode, Tosoh HLC-723 G11 variant/fast mode, Bio-Rad D-100, and Sebia Capillarys 2 Flex Piercing.

(A) Arkray ADAMS HA-8190V variant mode, (B) Arkray ADAMS HA-8190V fast mode, (C) Tosoh HLC-723 G11 variant mode, (D) Tosoh HLC-723 G11 standard mode, (E) Bio-Rad D-100, (F) Sebia Capillarys 2 Flex Piercing. Abbreviations: Hb, hemoglobin

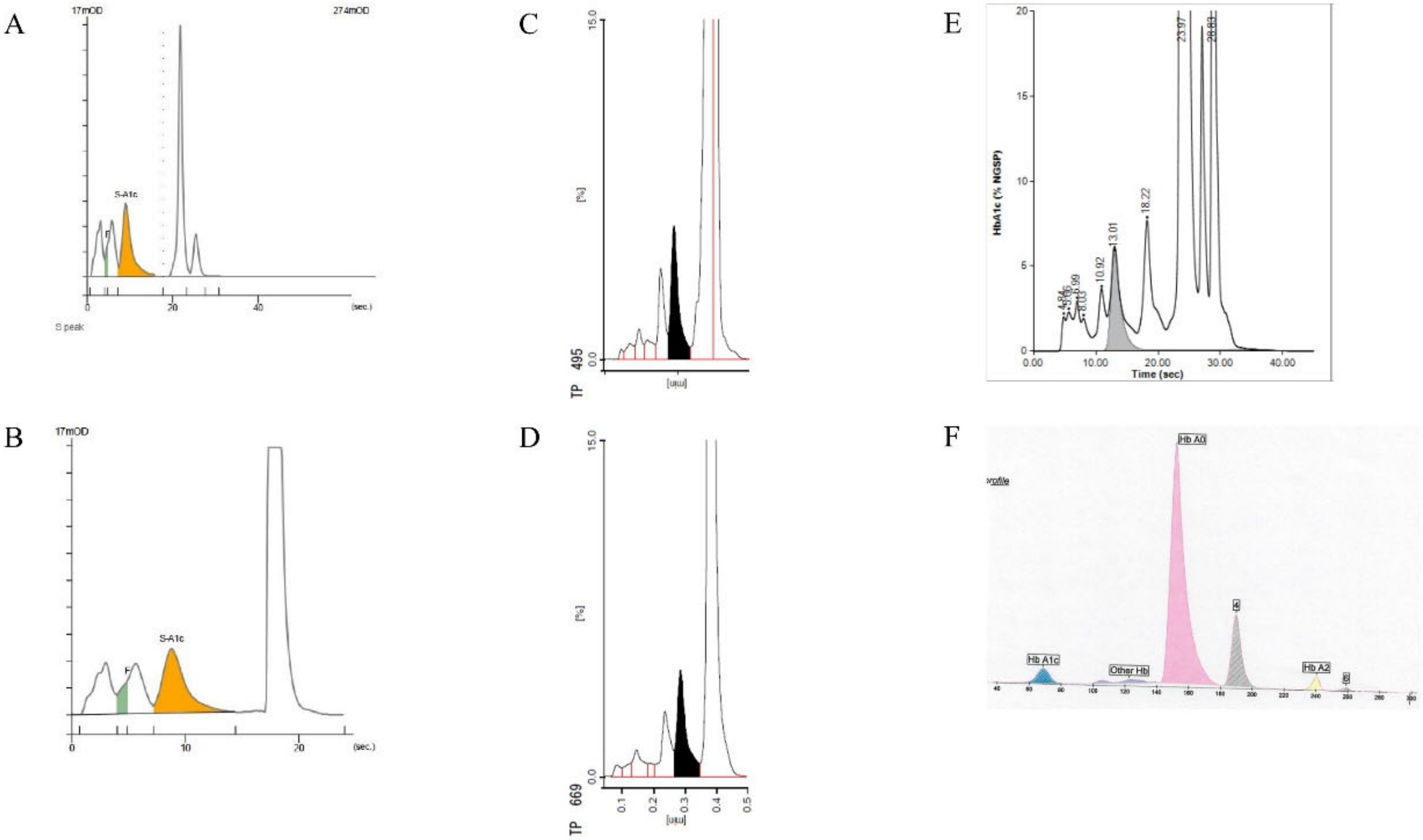
A
17mOD
274mOD
S-A1c
S peak
(sec.)
B
17mOD
S-A1c
(sec.)
C
15.0
[%]
0.0
TP 495
[mm]
D
15.0
[%]
0.0
TP 669
[mm]
E
HbA1c (% NGSP)
Time (sec)
23.97
28.83
18.22
13.01
10.92
F
Hb A0
Hb A1c
Other Hb
Hb A2

Supplementary Figure S3. The chromatograms or electropherograms of Hb Queens sample by Arkray HA-8190V variant/fast mode, Tosoh HLC-723 G11 variant/fast mode, Bio-Rad D-100, and Sebia Capillarys 2 Flex Piercing.

(A) Arkray ADAMS HA-8190V variant mode, (B) Arkray ADAMS HA-8190V fast mode, (C) Tosoh HLC-723 G11 variant mode, (D) Tosoh HLC-723 G11 standard mode, (E) Bio-Rad D-100, (F) Sebia Capillarys 2 Flex Piercing. Abbreviations: Hb, hemoglobin

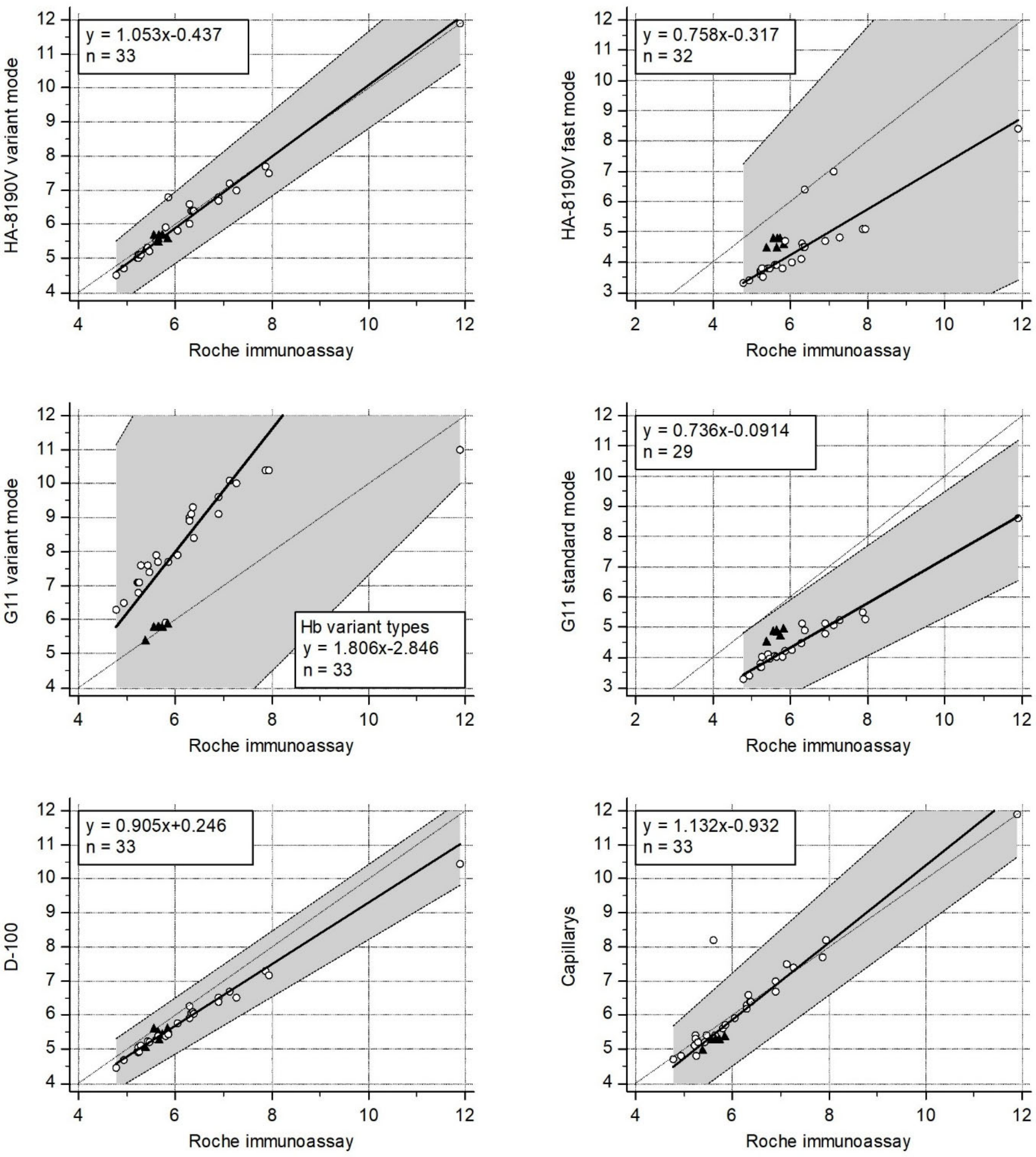

y = 1.053x-0.437
n = 33
HA-8190V variant mode
Roche immunoassay
y = 0.758x-0.317
n = 32
HA-8190V fast mode
Roche immunoassay
G11 variant mode
Hb variant types
y = 1.806x-2.846
n = 33
Roche immunoassay
y = 0.736x-0.0914
n = 29
G11 standard mode
Roche immunoassay
y = 0.905x+0.246
n = 33
D-100
Roche immunoassay
y = 1.132x-0.932
n = 33
Capillarys
Roche immunoassay

Supplementary Figure S4. Passing-Bablok regression analysis of HbA1c measurement methods in 33 samples with Hb G-Coushatta and Hb Queens variants.

Comparison of six different HbA1c measurement methods (Y-axis) against the Roche Tina-quant HbA1c Gen.3 immunoassay (X-axis) using clinical samples containing Hb Queens (n=7) and Hb Coushatta (n=26). The solid lines represent the Passing-Bablok regression lines, and the shaded areas indicate the 95% confidence intervals for the regression. The dashed lines represent the identity line (y = x). Open circles indicate samples with Hb G-Coushatta, and black triangles indicate those with Hb Queens. Hb variant types were confirmed using UPLC-MS/MS.

Abbreviations: HbA1c, Glycated Hemoglobin; UPLC-MS/MS, Ultra-Performance Liquid Chromatography–Tandem Mass Spectrometry; Roche immunoassay, The Roche Tina-quant HbA1c Gen. 3 immunoassay; HA-8190V, Arkray ADAMS HA-8190V (Arkray, Kyoto, Japan); G11, Tosoh HLC-723 G11 (Tosoh, Tokyo, Japan); D-100, Bio-Rad D-100 (Bio-Rad, Hercules, CA, USA); Capillarys, Sebia Capillarys 2 Flex Piercing (Sebia, Lisses, France).
